# The Double-Leucine Motifs Affect Internalization, Stability, and Function of Organic Anion Transporting Polypeptide 1B1

**DOI:** 10.3390/pharmaceutics15092279

**Published:** 2023-09-04

**Authors:** Xuyang Wang, Jieru Chen, Jiujiu Huang, Mei Hong

**Affiliations:** 1 College of Life Sciences, South China Agricultural University, Guangzhou 510642, China; 2 Guangdong Provincial Key Laboratory of Protein Function and Regulation in Agricultural Organisms, Guangzhou 510642, China

**Keywords:** OATP1B1, double-leucine motifs, intracellular loops, transmembrane helices, transport function

## Abstract

Organic anion transporting polypeptide 1B1 (OATP1B1) is specifically expressed at the basolateral membrane of human hepatocytes and plays important roles in the uptake of various endogenous and exogenous compounds including many drugs. The proper functioning of OATP1B1, hence, is essential for the bioavailability of various therapeutic agents and needs to be tightly regulated. Dileucine-based signals are involved in lysosomal targeting, internalization, and trans-Golgi network to endosome transporting of membrane proteins. In the current study, we analyzed the 3 intracellular and 13 transmembrane dileucine motifs (DLMs) within the sequence of OATP1B1. It was found that the simultaneous replacement of I332 and L333 with alanine resulted in a significantly reduced level of the mature form of OATP1B1. The cell surface expression of I332A/L333A could be partially rescued by MG132, as well as agents that prevent clathrin-dependent protein internalization, suggesting that this dileucine motif may be involved in the endocytosis of OATP1B1. On the other hand, I376/L377 and I642/L643, which are localized at transmembrane helices (TM) 8 and 12, respectively, are involved in the interaction of the transporter with its substrates. I642A/L643A exhibited a significantly decreased protein level compared to that of the wild-type, implying that the motif is important for maintaining the stability of OATP1B1 as well.

## 1. Introduction

Organic anion transporting polypeptides (OATPs) (gene symbol *SLCO*) are solute carrier (SLC) superfamily members and mediate sodium-independent transport of many structurally independent compounds [1]. Thus far, there are 12 human OATPs that have been identified. Some OATP members are ubiquitously expressed, while others are predominantly expressed in certain organs or tissues [2]. OATP1B1 is the major OATP that is selectively expressed at the basolateral membrane of human hepatocytes, and quite a few endogenous compounds and clinically important drugs are substrates of OATP1B1 [3]. Due to the broad substrate spectra, OATP1B1 has been well recognized to serve important roles in drug clearance from the body and is an important site for drug–drug interaction [4]. Therefore, the proper functioning of OATP1B1 is essential for the bioavailability of various therapeutic agents. 

Although information related to substrates transported by OATPs has been growing, studies concerning their cellular regulation are limited. After translation, membrane proteins often need to go through an orchestrated series of events to eventually target the plasma membrane, at which they exert their function [5]. On the other hand, proteins on the cell membrane are not static and may go through internalization, recycling, and/or degradation in a dynamic way. The targeting and sorting of membrane proteins have been demonstrated to be mediated by signals consisting of short, linear sequences of amino acid residues in the cytosolic domains of the proteins [6]. For example, NPxY (in which x can be any amino acid) is a kind of tyrosine-based sorting signal that is involved in the sorting [7] and endocytosis regulation [6] of membrane proteins. Our recent study revealed a novel role of the NPxY motif at the intracellular loop 3 (IL3) of OATP1B1. The asparagine and proline residues together are involved in regulating the exit of the transporter from the Golgi apparatus, while the tyrosine residue may be important for maintaining the conformation of OATP1B1 [8]. Another type of signal referred to as dileucine-based signals has been proposed to be involved in basolateral and lysosomal targeting, internalization, and trans-Golgi network to endosome sorting of membrane proteins [6]. For example, it was found that the dileucine motif (DLM) located at the third intracellular loop (IL) of rat sodium taurocholate co-transporting polypeptide (Ntcp) is important for the plasma membrane expression and endocytosis of the transporter [9]. WC1, a γδ T cell coreceptor, contains a dileucine endocytosis motif at its cytoplasmic domain, which is essential for the activation of the T cells [10]. Additionally, the double-leucine motif in transmembrane helix 12 was demonstrated to be important for the functioning of organic anion transporter 1 (OAT1). The mutant transporter L503/L504A was trapped in the endoplasmic reticulum and failed to be converted into the mature form, hence unable to exert its function on the plasma membrane [11]. 

**Figure 1:**
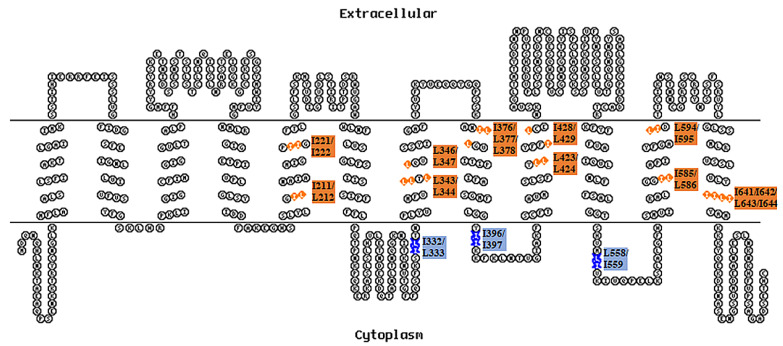
Location of different dileucine motifs within the sequence of OATP1B1. Amino acid residues along the transmembrane helices were selected according to the Kyte–Doolittle hydrophobicity scale. The topology model of OATP1B1 was generated with the web-based service TOPO2 (https://www.cgl.ucsf.edu/Overview/software.html#topo2 (accessed on 12 March 2020)). Dileucine motifs at intracellular loops are labeled as blue stars, while those at the transmembrane helices are indicated as orange diamonds.

OATP1B1 has a putative 12-transmembrane-helix (TM) topology according to computer-based hydropathy analysis. There are altogether 16 potential DLMs within the sequence of the transporter, with 3 localized intracellularly and 13 at the transmembrane helices (Figure 1). Our current study identified a DLM at IL3 that is involved in the internalization of OATP1B1 and two DLMs within the transmembrane helices that may affect the protein stability and function of the transporter. 

## 2. Materials and Methods

### 2.1. Materials

[^3^H]Estrone-3-sulfate (ES) was purchased from PerkinElmer Life Sciences (Waltham, MA, USA). Sulfosuccinimidyl 2-(biotinamido)-ethyl-1, 3-dithiopropionate (NHS-SS-biotin), and streptavidin-agarose beads were from Thermo Fisher Scientific (Waltham, MA, USA). All other reagents were obtained from Sigma (St. Louis, MO, USA), except otherwise stated. 

### 2.2. Site-Directed Mutagenesis

Site-directed mutagenesis was carried out with the QuikChange Lightning Site-Directed Mutagenesis Kit from Agilent (Santa Clara, CA, USA). The pReceiver M07 vector containing the *SLCO1B1* cDNA and 3-HA tags at the C-terminus (Genecopoeia, Rockville, MD, USA) served as the template for the mutagenesis. All mutant sequences were confirmed by full-length sequencing by Thermo Fisher Scientific.

### 2.3. Transfection of Plasmid Constructs

Human embryonic kidney cells (HEK293) were grown in Dulbecco’s modified Eagle’s medium (Thermo Fisher Scientific) supplemented with 10% fetal bovine serum. DNA plasmids were transfected into confluent cells with LipofectAMINE 2000 reagent (Thermo Fisher Scientific) following the manufacturer’s instruction. Transfected cells were incubated for 48 hrs at 37 °C and then used for the transport assay or protein analysis.

### 2.4. Uptake of Different Substrates by OATP1B1

Cells in 48-well plates were used for transport measurement. Briefly, cells were incubated with uptake solution containing [^3^H]ES or 2′, 7′-dichlorofluorescein (DCF) at 37 °C, and the transport process was stopped with ice-cold phosphate-buffered saline (PBS) solution. Cells were then washed with cold PBS, solubilized in NaOH before further detection with a liquid scintillation counter (Triathler-Hidex, Hidex, Turku, Finland) or a SpectraMax i3x Multi-Mode Microplate Reader (Molecular Devices, San Jose, CA, USA). The uptake count was standardized by the amount of protein in each well. 

### 2.5. Calculation of Additive Effect

The additive effect of two adjacent single-mutants was calculated based on the Bliss independence model [12] with the equation 1 − (Ma + Mb × (1 − Ma)), in which Ma and Mb represented the change of the uptake function caused by each mutant. Each single-mutant was supposed to act independently of the adjacent single-mutant. 

### 2.6. Biotin Labeling of Cell Surface Proteins and Western Blotting

The membrane-impermeable biotinylation reagent NHS-SS-biotin was utilized to examine the cell surface level of OATP1B1 and mutants. Briefly, HEK293 cells expressing OATP1B1 or mutants were labeled with 0.5 mg/mL of NHS-SS-biotin. After dissolving the cells with RIPA buffer, streptavidin-agarose beads were added to bind the biotin-labeled membrane proteins. The bound proteins were then released in Laemmli buffer, loaded onto the SDS-polyacrylamide electrophoresis gel, transferred electrophoretically to a polyvinylidene difluoride membrane (Millipore, Billerica, MA, USA), and finally, detected with anti-HA antibody (Beyotime Biotechnology, Jiangsu, China). Actin and integrin were used as loading controls for total and membrane proteins, respectively. Three independent experiments were performed for each analysis, and one representative blot is shown. 

### 2.7. Statistical Analysis

One-way analysis of variance (ANOVA) followed by Dunnett’s test was used for data comparison. Differences between groups were regarded as statistically significant if *p* < 0.05.

## 3. Results

### 3.1. Identification of Important Double-Leucine Motifs in the Intracellular Loops and Transmembrane Domains of OATP1B1

**Figure 2:**
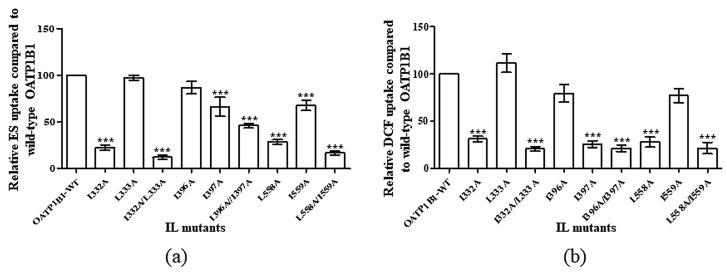
Uptake function of double-leucine mutants in the intracellular loops of OATP1B1. Uptake of 50 nM ES was measured at 37 °C at a 2 min interval (**a**) or 1 μM DCF was measured at 37 °C at a 5 min interval (**b**). Net uptake was obtained by subtracting the uptake of cells transfected with the empty vector from cells expressing OATP1B1 or mutants. Mutant uptakes are presented as the percentage of that of the wild-type transporter. The results represent data from three independent experiments, with duplicate measurements for each sample. The results shown are the means ± S.D. (*n =* 3). Asterisks indicate significantly different from wild-type OATP1B1 (*** *p* < 0.001).

To identify whether the two adjacent leucine and/or isoleucine exert their function as an integrated part, we first generated single-mutants and double-mutants of the corresponding motifs indicated in Figure 1. The transport function of the corresponding single-mutants and double-mutants was then measured with the prototypic OATP substrates estrone-3-sulfate (ES) and 2′, 7′-dichlorofluorescein (DCF). As shown in Figure 2a,b, most of the mutants exhibited significantly reduced uptake function for both ES and DCF, with the exception of L333A and I396A. Nevertheless, all double-mutants showed a more dramatic alteration of function compared to either of the corresponding single-mutants. To clarify whether the reduction observed in the double-mutant was merely an additive effect of the two single-mutants, we calculated the combined effect of two adjacent single-mutants, supposing that each of them exerted the influence independently. The remaining function derived from the additive effect of two single-mutants was then compared to that of the function of the corresponding double-mutant. As shown in Table 1, only the double-mutant I332A/L333A exhibited significantly reduced function compared to that derived from the calculated additive effect, suggesting that the DLM may act as an integrated motif for the function of OATP1B1.

**Figure 3:**
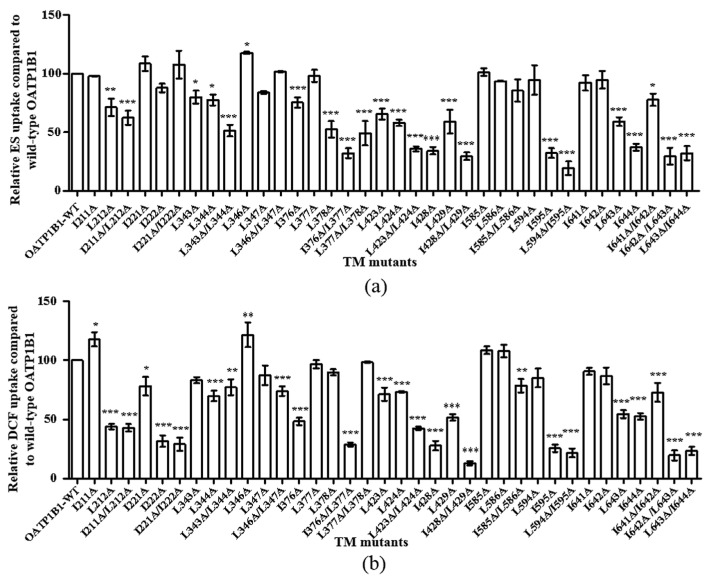
Uptake function of double-leucine mutants in the transmembrane domains of OATP1B1. Uptake of 50 nM ES was measured at 37 °C at a 2 min interval (**a**) or 1 μM DCF was measured at 37 °C at a 5 min interval (**b**). Net uptake was obtained by subtracting the uptake of cells transfected with the empty vector from cells expressing OATP1B1 or mutants. Mutant uptakes are presented as the percentage of that of the wild-type transporter. The results represent data from three independent experiments, with duplicate measurements for each sample. The results shown are the means ± S.D. (*n =* 3). Asterisks indicate significantly different from wild-type OATP1B1 (* *p* < 0.05, ** *p* < 0.01, *** *p* < 0.001).

The double-leucine motifs within the transmembrane domains were analyzed in a similar way. Although quite a few single- and double-mutants showed significantly reduced uptake function (Figure 3), only the activity of two DLMs, i.e., I376A/L377A and I642A/L643A, decreased significantly compared to the uptake function derived from the calculated additive effect of the corresponding single-mutants (Table 2 and Table 3). Moreover, the same trend was observed for both intracellular and transmembrane residues with the uptake of ES or DCF; hence, DCF was used for the uptake function analysis in our following studies. 

### 3.2. Protein Level Analysis of the Critical Double-Leucine Motifs in the Intracellular Loop

**Figure 4:**
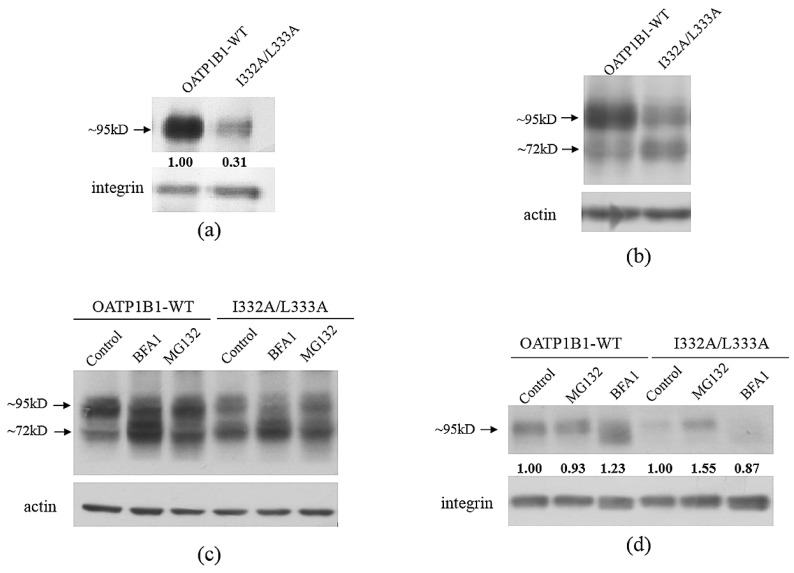
The protein expression of the I332A/L333A double-mutant. (**a**) Cell surface expression of OATP1B1 and I332A/L333A double-mutant. (**b**) Total protein expression of OATP1B1 and I332A/L333A. Cells expressing OATP1B1 wild-type or the mutant were biotinylated and lysed, and the biotin-labeled proteins were precipitated with streptavidin beads. In the case of total protein expression, cells were directly lysed with RIPA buffer. The proteins were then denatured and separated by SDS-PAGE, followed by Western blotting, and detected with anti-HA antibody (1:1000 dilution) or probed with integrin (membrane proteins) or actin (total proteins) antibodies as the loading control. (**c**) Total protein expression of OATP1B1 and I332A/L333A after the treatment with proteasome inhibitor MG132 or lysosome inhibitor BFA1. (**d**) Cell surface expression of OATP1B1 and I332A/L333A after MG132 or BFA1 treatment. Cells were treated with 10 μM MG132 or 100 nM BFA1 for 12 h, and the proteins were extracted and analyzed as described above. The intensity of the bands for cell surface transporter proteins was analyzed with Image J, adjusted with the loading control, and shown under the corresponding samples. The band intensity of wild-type OATP1B1 is designated as 1 in (**a**), while the band intensity of the untreated wild-type OATP1B1 or I332A/L333A is designated as 1 in (**d**).

As a membrane protein, OATP1B1 needs to be correctly targeted to the plasma membrane to exert its function. Therefore, the effect caused by these DLMs may be due to the inability of the mutants to express on the cell surface. As shown in Figure 4a, the I332A/L333A cell surface level was significantly reduced compared to that of OATP1B1-WT. However, when the total protein level of the double-mutant was analyzed, it was found that, though the mature form (~95 kD) was significantly reduced in the mutant, the immature form (~72 kD) level was higher in I332A/L333A compared to that of wild-type OATP1B1 (Figure 4b). 

Since membrane proteins are usually degraded through the proteasome or lysosome pathways [13], we, therefore, treated I332A/L333A with MG132, a proteasome inhibitor, and bafilomycin A1 (BFA1), a lysosome inhibitor. As shown in Figure 4c, though both MG132 and BFA1 treatments increased the immature form of the transporter, only the BFA1 treatment increased the mature form level of wild-type OATP1B1. On the other hand, the mature form of I332A/L333A was only partially recovered by the MG132 treatment. Moreover, when proteins on the cell surface were analyzed, I332A/L333A on the plasma membrane was elevated after MG132 treatment (Figure 4d). 

### 3.3. The I332/L333 Motif May Play a Role in Protein Internalization

**Figure 5:**
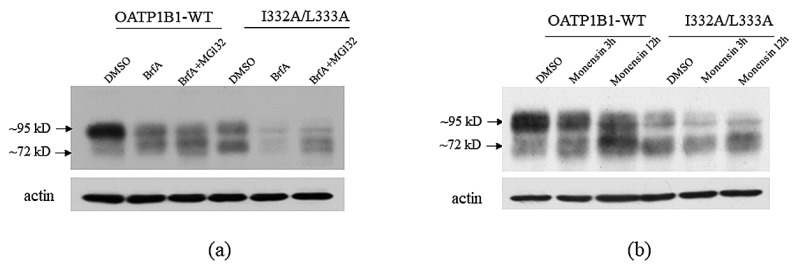
Treatment of I332A/L333A double-mutant with Golgi transport blockers. (**a**). Cells expressing the OATP1B1 or the I332A/L333A double-mutant were treated with Brefeldin A. (**b**). Cells expressing the OATP1B1 or the I332A/L333A double-mutant were treated with monensin. Cells expressing OATP1B1 or I332A/L333A were treated with 2 μM BrfA for 16 h or 1 μM monensin for 3 and 12 h. Total proteins were extracted and subjected to analysis.

After translation, membrane proteins need to go through a series of complicated events [5]. The newly synthesized polypeptide chains are firstly targeted to the endoplasmic reticulum (ER) to be checked for correct folding and glycosylated and then transported to the Golgi apparatus and, eventually, to the plasma membrane [14]. We currently demonstrated that an NPxY motif localized at intracellular loop 3 (IL3) of OATP1B1 is important for the transporter to exit the Golgi apparatus [8]. As I332A/L333A is also located at IL3, we speculated that the motif may play a similar role. However, when Brefeldin A (BrfA), a lactone antibiotic, which inhibits protein transport from the endoplasmic reticulum to the Golgi apparatus, was applied, the mature form of the transporter was reduced and the immature form was increased in both the wild-type transporter and the double-mutant. Although BrfA treatment resulted in a dramatic reduction of I332A/L333A, MG132 was able to recover the immature form of the transporter (Figure 5a). Treatment with monensin, a blocker that interferes with protein transport through the Golgi stack [15], also led to a similar response in OATP1B1-WT and I332A/L333A, i.e., the immature form of both transporters was increased at the expense of the mature form (Figure 5b). These results suggested that the I332/L333 motif may not be involved in the processing of OATP1B1 through the Golgi apparatus. 

**Figure 6:**
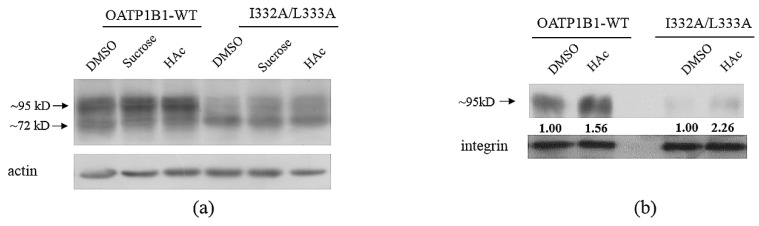
Internalization analysis of the I332A/L333A double-mutant. Cells were treated with 0.45 M sucrose for 30 min or with 5 mM acetic acid for 1 h. Total (**a**) and cell surface proteins (**b**) were extracted and analyzed as described in Figure 4. The intensity of the bands for cell surface transporter proteins was analyzed with Image J, adjusted with the loading control, and shown under the corresponding samples. The band intensity of the untreated wild-type OATP1B1 or I332A/L333A is designated as 1.

Studies have shown that DLMs may be involved in the internalization of membrane proteins, and OATP1B1 was demonstrated to be internalized through the clathrin-dependent pathway [16]; we, hence, explored this likely role of I332/L333 with the treatment with hypertonic sucrose or acidification of the cytoplasm with acetic acid (HAc). As shown in Figure 6a, both sucrose and HAc treatment led to an increased level of the mature form of I332A/L333A, and a less-significant effect was observed in the wild-type transporter. Cell surface analysis revealed a similar effect, with a significantly elevated level of I332A/L333A observed at the plasma membrane after HAc treatment (Figure 6b). 

### 3.4. Protein Level Analysis of the Critical Double-Leucine Motifs in the Transmembrane Domains

**Figure 7:**
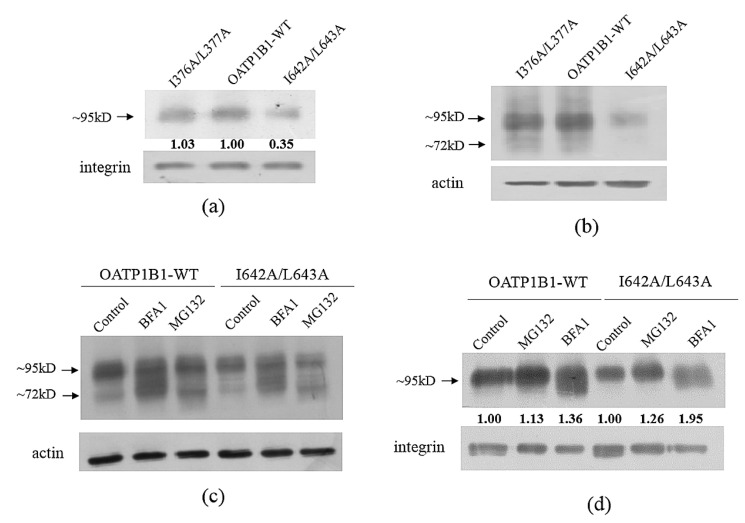
Analysis of the protein expression of TM mutants. (**a**) Cell surface expression of OATP1B1, I376A/L377A, and I642A/L643A. (**b**) Total protein expression of OATP1B1 and TM mutants. Total (**c**) and cell surface (**d**) protein expression of OATP1B1 and TM mutants after treatment with MG132 or BFA1. The cell surface and total proteins of TM mutants were extracted and analyzed as described in Figure 4. The intensity of the bands for cell surface transporter proteins was analyzed with Image J, adjusted with the loading control, and shown under the corresponding samples. The band intensity of wild-type OATP1B1 is designated as 1 in (**a**), while the band intensity of the untreated wild-type OATP1B1 or I642A/L643A is designated as 1 in (**d**).

Since I376A/L377A and I642A/L643A, two DLMs at the transmembrane helices, also exhibited significantly reduced activity compared to the transport function derived from the calculated additive effect of the corresponding single-mutants (Table 2 and Table 3), the protein expression of these two mutants was analyzed as well. As shown in Figure 7a,b, I642A/L643A displayed a reduced protein level both at the total and cell surface protein pool, while I376A/L377A exhibited comparable expression to that of the wild-type transporter. Treatment with proteasome and lysosome inhibitors revealed that both the MG132 and BFA1 treatments increased the cell surface protein level of I642A/L643A, though BFA1 exhibited a more-profound effect than MG132 (Figure 7c,d).

### 3.5. Kinetic Analysis of the Important Dileucine Mutants

**Figure 8:**
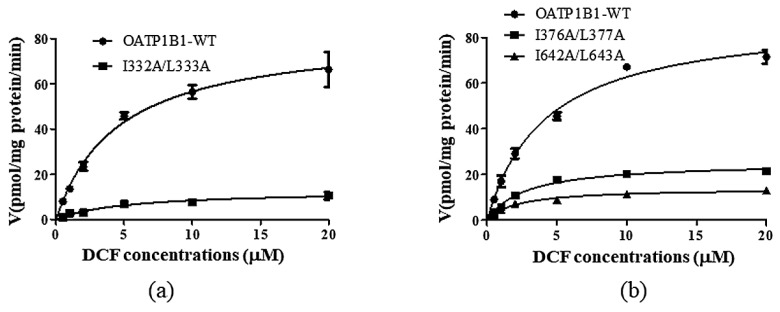
Kinetic analysis of dileucine mutants. The uptake of DCF was measured at concentrations ranging from 0.5 to 20 μM at 37 °C for 2 min. Net uptake was obtained by subtracting the uptake of cells with the empty vector from cells expressing wild-type OATP1B1 or I332A/L333A (**a**) or TM mutants (**b**). The results represent data from three independent experiments, with duplicate measurements for each sample. The results shown are the means ± S.D. (*n ≥* 3).

Although the change of the protein level on the cell surface may explain the dramatically reduced uptake by I332A/L333A and I642A/L643A, the mutation of these motifs may also alter the interaction of the transporter with the substrates; hence, we investigated the concentration-dependent transport of DCF by these mutants and found that I332A/L333A only affected the Vmax of the transporter (Figure 8a). However, when the function of the double-mutant was normalized with its cell surface protein level, the Vmax of the mutant was similar to that of OATP1B1-WT (Table 4), suggesting that the reduced function of the mutant was mainly due to the decreased level of the transporter. As for the DLMs at the TMs, both I376A/L377A and I642A/L643A exhibited a reduced Km (though not statistically significant for I376A/L377A) and significantly decreased Vmax (Figure 8b and Table 4). Moreover, even when the Vmax of I642A/L643A was normalized with the membrane protein level of the mutant, the value of the turn-over rate was still significantly lower than that of the wild-type transporter (Table 4), which indicated that this DLM not only affects the stability of OATP1B1, but also is involved in the interaction between the transporter and the substrates. When the overall transport efficiency was characterized by Vmax/Km, it was shown that all three mutants exhibited decreased transport efficiency compared to that of wild-type OATP1B1, with I376A/L377A exhibiting the most-dramatic reduction (Table 4). 

## 4. Discussion

OATP1B1 is specifically expressed at the sinusoidal membrane of hepatocytes, playing an essential role in the uptake of a wide range of substrates, including many drugs. As a membrane protein, it is dynamically processed within the cells, responding to different cues for exerting its functions, and hence, needs to be precisely regulated. 

The double-leucine motif has been reported to serve as a recognition site for cellular processing such as lysosomal targeting, internalization, and trans-Golgi network to endosome transporting of various membrane proteins [7,17]. Within the sequence of OATP1B1, there are three potential intracellular DLMs, and it was identified that I332/L333, which is localized in the large IL3 between TM5 and TM6, plays an important role in maintaining the mature form level of the transporter. A comparison of the additive effect of the uptake function of I332A and L333A with the effect that was caused by the I332/L33A double-mutant revealed that the two consecutive leucine/isoleucine residues may exert the function as an integral motif. Further investigation showed that the double-mutant exhibited significantly reduced total and cell surface protein expression, especially for the mature form of the transporter protein. Our previous study identified that an NPxY motif, which is also localized within IL3, is important for the exit of OATP1B1 from the Golgi apparatus [8]. Hence, the ER and Golgi blockers were applied to treat I332A/L333A, and the responses were analyzed. It was found that I332A/L333A exhibited a similar response to the blockers compared to wild-type OATP1B1, implying that the mutation of the motif did not affect the processing of the protein through the ER and Golgi apparatus. On the other hand, the reduction in the mature form of OATP1B1 may be due to an accelerated internalization and degradation of the transporter. It was previously demonstrated that OATP1B1 may go through the clathrin-dependent endocytosis pathway [16]. When the cells expressing the double-mutant I332A/L333A were treated with sucrose and acetic acid, both of which prevent the clathrin-dependent endocytosis process of membrane proteins, there was a significant increase of the mature form of the protein observed, suggesting that the DLM may be involved in the endocytosis of the transporter. Internalized membrane proteins are usually degraded through the endosome–lysosome pathway [18]. However, it was found in our current study that the reduced protein level in I332A/L333A was partially rescued by the proteasome inhibitor MG132, but not the lysosome inhibitor BFA1, suggesting that the internalized mutant may be degraded by the proteasome instead of the lysosome. Degradation of internalized proteins through the proteasome pathway was reported in the metal transporter Zrt/IRT-like protein 14 (ZIP14). ZIP14 was first extracted from the plasma membrane, then deglycosylated and targeted to proteasome degradation in response to iron deficiency. It was suggested that retro-translocation machinery and glycosylation are required for endocytosis and the subsequent degradation of ZIP14. Unglycosylated ZIP4 failed to internalize and is more stable than the glycosylated form [19]. Interestingly, the less-glycosylated immature form of I332A/L333A also seemed to be more stable and exhibited a higher level than wild-type OATP1B1 in our current study. Whether the I332/L333 motif linked to the glycosylation of OATP1B1 and together regulate the extraction of the transporter from the cell surface in response to certain extracellular stimuli is the subject of future investigations. Moreover, the kinetic analysis suggested that I332/L333 is not involved in substrate binding and/or recognition of the transporter, since both the Km and Vmax values are similar to that of the wild-type transporter when the cell surface protein level was taken into consideration. Therefore, the intracellular DLM seems to only play a regulatory role for the cellular processing of OATP1B1, but not in the direct function of the transporter. 

Double-leucine motifs within the transmembrane helices, on the other hand, have been reported to affect the uptake function of transporters such as OAT1 [11]. Our present study also found that the DLMs in TM8 and TM12 may play important roles in the substrate transport of OATP1B1. It was found that the decreased expression of I642A/L643A could be partially rescued by both proteasome and lysosome inhibitors, suggesting that, though the mutation of I332/L333 and I642/L643 both resulted in accelerated protein degradation, the two DLMs may exert their effect in different ways. Kinetic analysis demonstrated that the Km and Vmax values of I642A/L643A were significantly reduced compared to those of wild-type OATP1B1, indicating that the DLM not only plays a role in maintaining the protein stability, but is also involved in the interaction between the transporter and the substrates. However, it should be pointed out that, when the transport efficiency of I642A/L643A was characterized by Vma/Km using the Vmax value that was adjusted with the relative cell surface level of the mutant, the ratio was much higher than that of I376A/L377A, suggesting that the reduced uptake function observed in I642A/L643A is mainly due to its much-reduced protein expression. It is interesting to note that I642/L643 is located in the middle of a four-leucine/-isoleucine region (641–644). However, the double-mutation of I641 and I642 or L643 and I644 did not result in a synergetic effect. Moreover, the simultaneous mutation of all four residues resulted in a comparable effect to that calculated from the combination of four individual mutants, suggesting that only the two leucine/isoleucine residues in the middle form an integral motif to regulate the transporter protein. Further study on this specific transmembrane helix is currently underway in our laboratory. On the other hand, I376A/L377A, which is localized in TM8, only showed a marginal change in the protein level, but a significantly reduced Vmax for the transport of DCF. The Km value of the double-mutant showed a decreasing trend, but was not significantly different compared to that of the wild-type OATP1B1. Previous studies have demonstrated that TM8 is critical for substrate recognition of OATP1B1. When the transmembrane helix in OATP1B1 was substituted by that of OATP1B3, the uptake function of OATP1B1 for both estrone-3-sulfate and estradiol-17beta-D-glucuronide was greatly reduced [20]. The reduction of both the Km and Vmax values of I376A/L377A suggested that the motif may be involved in regulating the interaction of the transporter with its substrates and that the mutation of this DLM resulted in the formation of a more-stable transporter–substrate complex. It is interesting to note that, though both OATP1B1 and 1B3 share a leucine residue at position 377, the corresponding residue in OATP1B3 for I376 in OATP1B1 is a phenylalanine. Such a replacement not only disrupts the integral DLM, but the bulkier side chain in phenylalanine also likely hinders the uptake function of the transporter. Further investigation is warranted to clarify the issue. 

## 5. Conclusions

In summary, our present study identified three important dileucine motifs within the sequence of OATP1B1. I332/L33 may play a role in regulating the endocytosis of OATP1B1. I376/L377 and I642A/L643A are likely involved in the interaction between the transporter and its substrates. Moreover, I642/L643 may also play a role in maintaining the stability of OATP1B1. However, further investigation is needed to clarify at which step(s) of the internalization the I332/L333 motif is (are) involved and how the DLM regulates the internalization process of OATP1B1. Further, since both I376/L377 and I642/L643 are important for the interaction of the transporter with its substrates and diverse combinations are observed at the corresponding locations of other OATP family members, it will be of interest to evaluate their roles in substrate recognition for different OATPs as well.

## Figures and Tables

**Table 1:** Comparison of the additive effect of two single-leucine/-isoleucine mutants with the corresponding double-leucine mutants in the intracellular loops of OATP1B1.

Substrate	ES			DCF		
	I332A/L333A	I396A/I397A	L558A/I559A	I332A/L333A	I396A/I397A	L558A/I559A
Additive effect	21.7 ± 5.5	52.9 ± 14.4	19.4 ± 5.0	37.8 ± 6.5	11.9 ± 3.6	22.5 ± 10.7
Double-mutants	12.3 * ± 4.1	64.8 ± 22.5	16.8 ± 3.6	16.6 * ± 6.4	12.3 ± 6.2	17.4 ± 12

The additive effect was calculated with the equation 1 − (Ma + Mb × (1 − Ma)) and presented as a percentage. Ma and Mb represent the reduced ratio of the uptake function of each mutant compared to that of wild-type OATP1B1, which is designated as 1. The uptake of double-mutants is presented as the percentage of that of the wild-type transporter. Asterisks indicate significantly different from the calculated additive effect (* *p* < 0.05).

**Table 2:** Comparison of the additive effect of two single-leucine/-isoleucine mutants with the corresponding double-leucine mutants in the transmembrane domains of OATP1B1 (ES as the substrate).

	I211A/ L212A	I221A/ I222A	L343A/ L344A	L346A/L347A	I376A/ L377A	L377A/ L378A	L423A/ L424A	I428A/ L429A	I585A/ L586A	L594A/ I595A	I641A/ I642A	I642A/ L643A	L643A/ I644A
Additive effect	64.8 ± 6.8	95.1 ± 6.8	62.8 ± 15.5	98.6 ± 3.6	73.3 ± 1.5	50.9 ± 16.4	38.4 ± 8.5	19.6 ± 2.5	94.9 ± 4.0	29.7 ± 2.5	81.0 ± 8.9	53.6 ± 10	22.3 ± 3.7
Double-mutants	62.5 ± 10.5	108 ± 21	51.4 ± 10.1	102 ± 1	32.2 * ± 8.4	49.2 ± 17.9	35.9 ± 3.9	29.6 ± 5.7	85.5 ± 13	19.3 ± 10	77.8 ± 10.0	30.0 * ± 12	32.2 ± 10.1

The additive effect was calculated with the equation 1 − (Ma + Mb × (1 − Ma)) and presented as a percentage. Ma and Mb represent the reduced ratio of the uptake function of each mutant compared to that of wild-type OATP1B1, which is designated as 1. The uptake of double-mutants is presented as the percentage of that of the wild-type transporter. Asterisks indicate significantly different from the calculated additive effect (* *p* < 0.05).

**Table 3:** Comparison of the additive effect of two single-leucine/isoleucine mutants with the corresponding double-leucine mutants in the transmembrane domains of OATP1B1 (DCF as the substrate).

	I211A/ L212A	I221A/ I222A	L343A/ L344A	L346A/L347A	I376A/ L377A	L377A/ L378A	L423A/ L424A	I428A/ L429A	I585A/ L586A	L594A/ I595A	I641A/ I642A	I642A/ L643A	L643A/ I644A
Additive effect	52.3 ± 10.7	25.0 ± 7.9	58.6 ± 10.6	108 ± 32	43.6 ± 8.1	87.5 ± 7.9	52.5 ± 6.4	12.6 ± 2.6	116 ± 4	22.4 ± 7.1	78.9 ± 11.4	49.3 ± 7.0	29.0 ± 6.6
Double-mutants	43.0 ± 7.8	29.1 ± 9.7	77.4 ± 13.3	74 ± 6.9	27.4 * ± 5.6	99.0 * ± 2.4	42.6 ± 2.3	13.0 ± 2.4	78.7 * ± 9.9	21.7 ± 6.4	72.9 ± 13.9	19.7 * ± 7.4	23.3 ± 8.5

The additive effect was calculated with the equation 1 − (Ma + Mb × (1 − Ma)) and presented as a percentage. Ma and Mb represent the reduced ratio of the uptake function of each mutant compared to that of wild-type OATP1B1, which is designated as 1. The uptake of double-mutants is presented as the percentage of that of the wild-type transporter. Asterisks indicate significantly different from the calculated additive effect (* *p* < 0.05).

**Table 4:** Kinetic parameters of DCF transported by OATP1B1 and the double-leucine mutants.

	Km (μM)	Vmax (pmol/mg Protein/min)	Vmax/Km
OATP1B1-WT	4.29 ± 0.67	87.9 ± 11.7	20.5
I332A/L333A	5.20 ± 1.72	74.1 ± 9.9 (normalized with protein from 16.2 ± 4.5)	14.2
I376A/L377A	3.38 ± 0.82	29.6 * ± 2.9 (normalized with protein from 26.6 ± 4.0)	8.76
I642A/L643A	2.16 * ± 0.34	37.4 * ± 4.3 (normalized with protein from 14.0 ± 2.3)	17.3

The uptake of DCF was measured at concentrations ranging from 0.5 to 20 μM at 2 min intervals. The measurements were then normalized with the relative cell surface level of the transporters. The nonlinear regression of the Michaelis–Menten equation incorporated in GraphPad Prism was performed to obtain the transporter kinetic parameters. The results shown are the means ± S.D. (*n* ≥ 3). Asterisks indicate values significantly different (*p* < 0.05) compared to that of wild-type OATP1B1.

## Data Availability

The data presented in this study are openly available in FigShare at DOI:10.6084/m9.figshare.24080499.

## References

[1] Zhang Y., Hagenbuch B. (2019). Protein-protein interactions of drug uptake transporters that are important for liver and kidney. Biochem. Pharmacol..

[2] Nakanishi T., Tamai I. (2012). Genetic polymorphisms of OATP transporters and their impact on intestinal absorption and hepatic disposition of drugs. Drug Metab. Pharmacokinet..

[3] König J. (2011). Uptake transporters of the human OATP family: Molecular characteristics, substrates, their role in drug-drug interactions, and functional consequences of polymorphisms. Handb. Exp. Pharmacol..

[4] Stieger B., Hagenbuch B. (2014). Organic anion transporting polypeptides. Curr. Top. Membr..

[5] High S., Laird V. (1997). Membrane protein biosynthesis—All sewn up?. Trends Cell Biol..

[6] Bonifacino J.S., Traub L.M. (2003). Signals for sorting of transmembrane proteins to endosomes and lysosomes. Annu. Rev. Biochem..

[7] Stoops E.H., Caplan M.J. (2014). Trafficking to the apical and basolateral membranes in polarized epithelial cells. J. Am. Soc. Nephrol..

[8] Wang X., Liang Y., Fang Z., Huang J., Hong M. (2019). The intracellular NPxY motif is critical in maintaining the function and expression of human organic anion transporting polypeptide 1B1. Biochim. Biophys. Acta Biomembr..

[9] Stross C., Kluge S., Weissenberger K., Winands E., Häussinger D., Kubitz R. (2013). A dileucine motif is involved in plasma membrane expression and endocytosis of rat sodium taurocholate cotransporting polypeptide (Ntcp). Am. J. Physiol. Gastrointest. Liver Physiol..

[10] Hsu H., Baldwin C.L., Telfer J.C. (2015). The endocytosis and signaling of the gammadelta T cell coreceptor WC1 are regulated by a dileucine motif. J. Immunol..

[11] Hong M., Li S., Zhou F., Thomas P.E., You G. (2010). Putative transmembrane domain 12 of the human organic anion transporter hOAT1 determines transporter stability and maturation efficiency. J. Pharmacol. Exp. Ther..

[12] Bliss C.I. (1939). The toxicity of poisons applied jointly. Ann. Appl. Biol..

[13] Ciechanover A. (2005). Proteolysis: From the lysosome to ubiquitin and the proteasome. Nat. Rev. Mol. Cell Biol..

[14] Bruce A., Johnson A., Lewis J., Raff M., Roberts K., Walter P., Anderson M., Granum S. (2007). Intracellular vesicular traffic. Molecular Biology of the Cell.

[15] Rosa P., Mantovani S., Rosboch R., Huttner W.B. (1992). Monensin and brefeldin A differentially affect the phosphorylation and sulfation of secretory proteins. J. Biol. Chem..

[16] Hong M., Hong W., Ni C., Huang J., Zhou C. (2015). Protein kinase C affects the internalization and recycling of organic anion transporting polypeptide 1B1. Biochim. Biophys. Acta.

[17] Peden A.A., Park G.Y., Scheller R.H. (2001). The Di-leucine motif of vesicle-associated membrane protein 4 is required for its localization and AP-1 binding. J. Biol. Chem..

[18] MacGurn J.A., Hsu P.C., Emr S.D. (2012). Ubiquitin and membrane protein turnover: From cradle to grave. Annu. Rev. Biochem..

[19] Zhao N., Zhang A.S., Worthen C., Knutson M.D., Enns C.A. (2014). An iron-regulated and glycosylation-dependent proteasomal degradation pathway for the plasma membrane metal transporter ZIP14. Proc. Natl. Acad. Sci. USA.

[20] Miyagawa M., Maeda K., Aoyama A., Sugiyama Y. (2009). The eighth and ninth transmembrane domains in organic anion transporting polypeptide 1B1 affect the transport kinetics of estrone-3-sulfate and estradiol-17beta-D-glucuronide. J. Pharmacol. Exp. Ther..

